# Prenatal Sonographic Clues to Diagnose External Genital Anomalies: Is It a Girl or a Boy?

**DOI:** 10.7759/cureus.46878

**Published:** 2023-10-11

**Authors:** Gokce Annac

**Affiliations:** 1 Department of Radiology, Medical Park Gebze Hospital, Kocaeli, TUR

**Keywords:** genital anomalies, ultrasound, prenatal, hypospadias, clitoromegaly

## Abstract

Introduction

The purpose of this study was to present the prenatal sonographic findings of external genital anomalies and determine diagnostic clues.

Methodology

In a single-center retrospective study, a total of 15,320 pregnant women underwent a routine ultrasound (US) screening between 18 and 40 weeks of gestation from March 2015 to January 2022. The fetuses with indeterminate sex and suspected genital anomalies were enrolled in the study. B-mod and three-dimensional (3D) imaging of the external genital organs were performed according to a local protocol in cases of genital anomalies. Prenatal and postnatal data were retrieved from the electronic health records.

Results

A total of 88 fetuses were included in the study. The prevalence of external genital anomalies was found to be 0.6%, and the degree of correspondence (DC) between prenatal and postnatal diagnoses of external genital anomalies was 94.3%. The most common genital anomaly was hypospadias with a frequency of 59%. Severe hypospadias was detected in five of six cases with chordee where the penoscrotal angle was below 30°. Approximately 70% of clitoromegaly cases with labial hypertrophy had a *horseshoe sign* on 3D images.

Conclusions

The DC between prenatal and postnatal diagnoses of external genital anomalies is high in this study. The novel diagnostic clues, such as *horseshoe sign* and *penoscrotal angle* may be useful in diagnosing and determining the severity of the external genital anomalies.

## Introduction

Determination of fetal sex has become a part of routine prenatal sonographic examinations. Various sonographic markers of male and female external genitalia have been determined in early and late pregnancy. Although fetal sex determination is possible in most pregnancies, it can be difficult in some cases, and it may be possible to misidentify external genital organs. Many genital anomalies, such as hypospadias and clitoromegaly, may confuse fetal sex determination.

Hypospadias is a male genitourinary anomaly in which the embryological urethral groove fails to close [1]. Therefore, the position of the urethral canal becomes abnormal along the length of the ventral shaft of the penis, scrotum, or perineum. The failed embryological closure is often associated with chordee (curvature of the penis with ventral shortening) [2]. Hypospadias is the most common congenital sexual anomaly in males with a prevalence of 2.09/1000 live births [3]. On sonographic examination, a small incurved penis with a blunt-ended tip between the scrotal folds may raise suspicion for hypospadias. This view of the external genitalia was similar to the *tulip sign *previously mentioned by Meizner [4]. The appearance of hypospadias may mimic female external genitalia or can be interpreted as ambiguous genitalia or clitoromegaly. Fetal clitoromegaly is a female genital anomaly that is usually caused by maternal and fetal hormonal disorders, hormone therapy, or maternal exposure to chemicals as well as unexplained conditions [5]. Despite improvement in ultrasound (US) technology and specialization in the fetal US, the detection of genital anomalies remains inaccurate compared to other congenital anomalies [6].

An accurate diagnosis of genital anomalies may be of high value to clinicians and parents by providing counseling and allowing early management of associated anomalies and syndromes. This study aims to present the prenatal sonographic findings of genital anomalies, determine diagnostic clues, and avoid pitfalls in fetal sex determination.

## Materials and methods

Pregnant women who attended a secondary referral center specialized in maternal healthcare between March 2015 and January 2022 were enrolled in this single-center retrospective study. A total of 15,320 pregnant women underwent a routine US screening between 18 and 40 weeks of gestation, and fetuses with indeterminate sex and suspected genital anomalies were enrolled in the study. The fetuses who were not able to be followed up postnatally and those with bladder extrophies were excluded from the study. US screenings were performed by the same radiologist expert in the obstetric US using a high-resolution US device with a convex 6- to 1.9-MHz probe (Toshiba Aplio 500, Tokyo, Japan). Written informed consent was obtained from all parents, and the study was approved by the local ethics committee (Institutional Review Board of Istinye University, 23/175).

**Figure 1:**
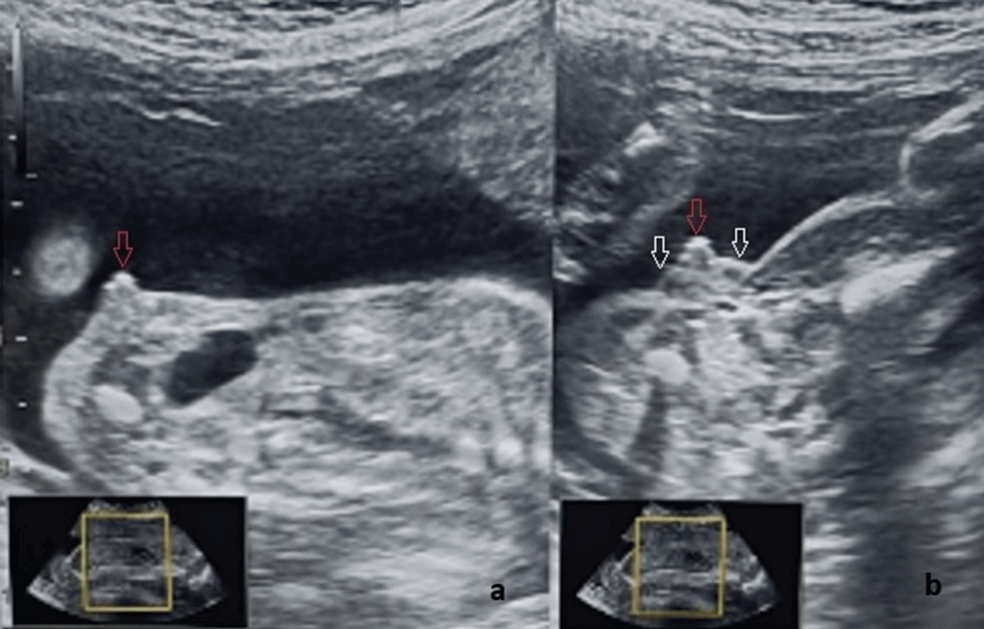
Clitoromegaly with labial hypertrophy (25 weeks of gestation). (a) Sagittal view of the enlarged clitoris (red arrow). (b) Axial view. Note the enlarged clitoris (red arrow) between two labial swellings (white arrows).

In case of uncertainty of the fetal sex, genital anatomical data focused on the configuration of the genital tubercle, position of the genital folds, and visualization of the pelvic organs. Clitoromegaly was suggested by visualization of a thickened and enlarged clitoris between two and four swellings representing the labia (Figure 1).

**Figure 2:**
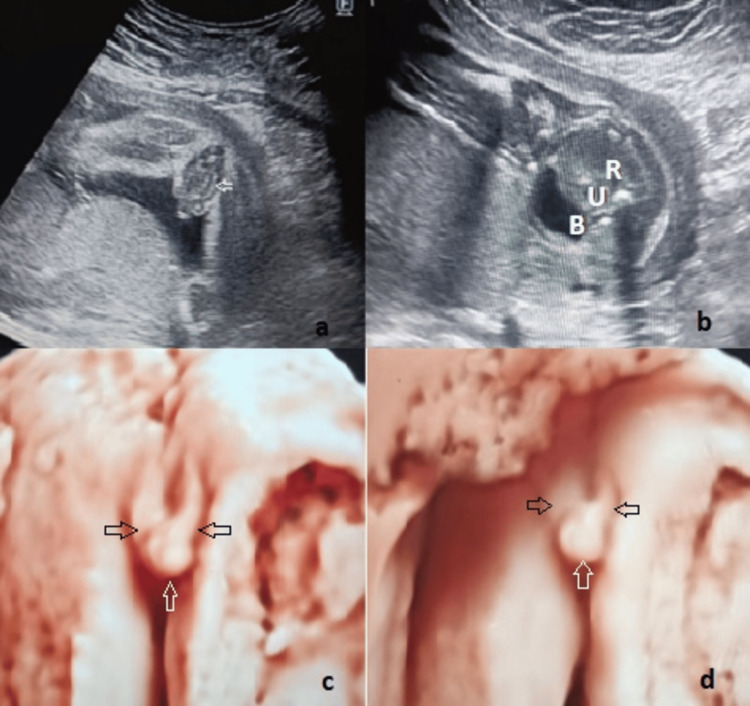
Clitoromegaly with labial hypertrophy (22 weeks of gestation). (a) Enlarged labia and a thickened clitoris. Note the concave line (white arrow) representing an enlarged labium minus. (b) Axial image of the pelvic region. Urinary bladder (B), uterus (U), and rectum (R) are followed from anterior to posterior, respectively. (c) and (d) 3D images of clitoromegaly (white arrow) and enlarged labia (black arrow). The concavity of both labia creates the appearance of a *horseshoe*.

Pelvic organs were evaluated in detail to make an accurate fetal sex determination. Demonstration of the uterus between the bladder and the rectum in the axial section of the pelvic region confirmed the diagnosis of the female gender. During routine sonographic examination, if any genital anomaly was suspected, it was considered abnormal and recorded as B-mod and also three-dimensional (3D) images as a local protocol for better evaluation of the anomaly (Figure 2).

**Figure 3:**
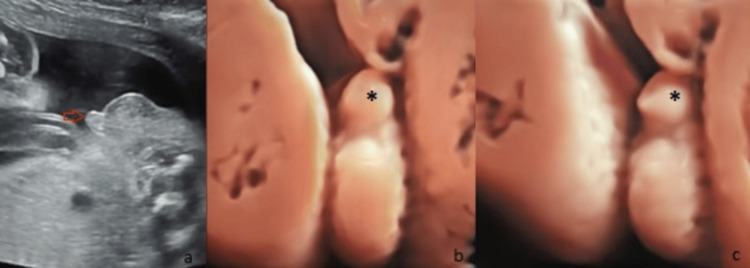
Hypospadias. Twenty-two weeks of gestation. (a) Sagittal image of a small penile shaft ending with a blunt tip (red arrow). (b) and (c) 3D images of hypospadias. Note the penis (*) with a blunt-ended tip.

**Figure 4:**
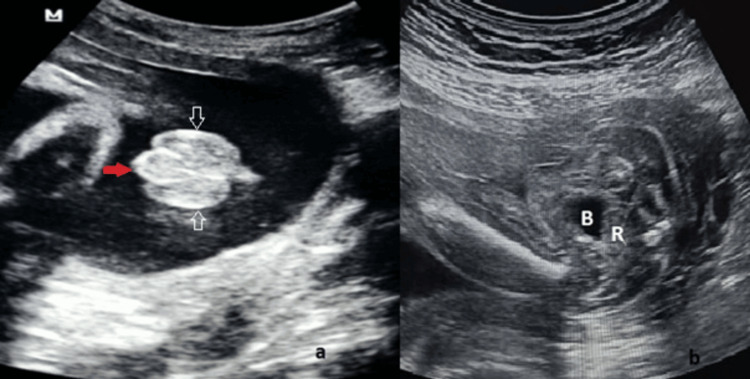
Severe hypospadias. Twenty-four weeks of gestation. (a) A small incurved penis (red arrow) with a blunt-ended tip between two scrotal folds (white arrows), which is called the *tulip sign*. (b) The rectum (R) is located just behind the bladder (B).

On sonographic examination, a small incurved penis with a blunt-ended tip between the scrotal folds was diagnosed as hypospadias (Figures 3-4). In the axial view of the pelvis, if the rectum was located just behind the bladder and there was no space between the bladder and the rectum, the diagnosis of male gender was confirmed (Figure 4b).

**Figure 5:**
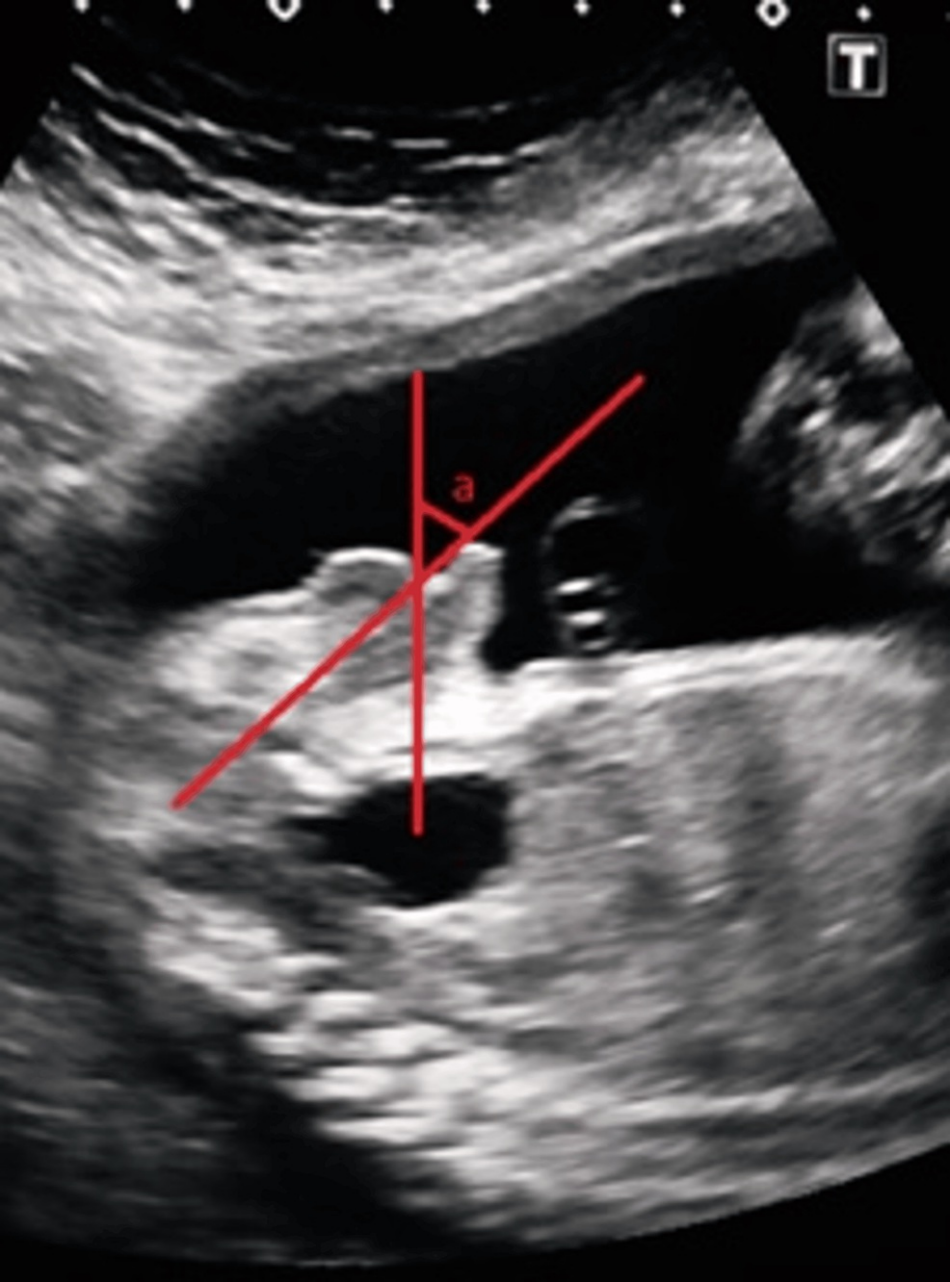
Measurement of the penoscrotal angle. Distal hypospadias with chordee, 23 weeks of gestation. Letter *a* represents the penoscrotal angle between the line extending from the ventral tip of the penis to the point where the scrotum meets the penis and the line passing from this point through the most anterior-superior part of the scrotum.

An angle between the line extending from the ventral tip of the penis to the point where the scrotum meets the penis and the line passing from this point through the most anterior-superior part of the scrotum were measured to evaluate the severity of the hypospadias with chordee. This angle was called the *penoscrotal angle*, which is shown as the letter *a* in Figure 5. Fetal adrenal size was evaluated according to a nomogram [7] to exclude adrenogenital syndrome.

Prenatal data were collected from the electronic health records of the study center’s obstetrics and gynecology department. History of genital anomalies in the family, exposure to chemicals, hormone therapy, associated anomalies, and integrated risk of aneuploidy were noted.

Postnatal data about infants with external genital anomalies were retrieved from the electronic health records of the pediatric and urology clinics. The demographic data of the patients, including hormonal study, molecular analyses, and clinical outcomes of the fetuses, were retrieved from the health records.

IBM SPSS Statistics for Windows, Version 22.0 (IBM Corp., Armonk, NY) was used for statistical evaluations. The Shapiro-Wilk test was used to check the normality assumption of the variables. Descriptive statistics were used as number, median, mean, frequency, and standard deviation (SD). The degree of correspondence (DC) was used to quantify the degree of resemblance between two sets of data. The DC between prenatal and postnatal diagnoses of the genital anomalies was calculated with the following formula, and it is expressed as a percentage: DC = number of correspondence data/number of valid data x 100. *P* < 0.05 was considered statistically significant for all tests.

## Results

Out of 15,320 fetuses, 92 (0.6%) fetuses with genital defects were detected on prenatal ultrasound. Four of them were excluded from the study due to a lack of postnatal data. The characteristics of the fetuses included in the study are given in Table 1.

**Table 1:** The characteristics of the fetuses included in the study. Maternal-fetal demographic characteristics, associated anomalies, and genetic syndromes are summarized. Data are represented as number (*N*) and mean ± SD. min, minimum; max, maximum; CNS, central nervous system; SD, standard deviation

Maternal-Fetal demographic characteristics		Value (min-max)
Maternal age	Years	25 ± 2 (19-40)
Gestational age at diagnosis	Weeks	19.5 ± 2 (18-27)
Associated anomalies/*N* (%)	Urogenital anomalies	7 (7.9)
	Extremity anomalies	3 (3.4)
	Cardiac anomalies	2 (2.2)
	CNS anomalies	1 (1.1)
	Abdominal wall defect	1 (1.1)
Genetic syndromes/*N* (%)	Trisomy 21	1 (1.1)

Of the 88 fetuses meeting the study’s inclusion criteria, prenatal ultrasound suggested a hypospadias diagnosis in 52 (59%) cases, clitoromegaly in 21 (23.8%, 17 with labial hypertrophy and four isolated), micropenis in seven (7.9%), isolated labial hypertrophy in five (5.6%), and uncertainty of sex determination in three (3.4%) cases. Of the 52 cases diagnosed with hypospadias in utero, 48 (​​92.3%) were confirmed at postnatal examination. Of these cases, 35 (72.9%) were classified as proximal/scrotal and 13 (27%) were glandular/distal hypospadias. Of 52 hypospadias cases, four (7.6%) were diagnosed with micropenis after birth. All of the cases diagnosed with clitoromegaly (*N *= 21 ), micropenis (*N *= 7), and isolated labial hypertrophy (*N *= 5) in utero were confirmed at postnatal examination (Table 2).

**Table 2:** Prenatal and postnatal diagnoses of genital anomalies. A comparison between prenatal and postnatal diagnoses of genital defects has been presented using numbers (*N*) and percentages (%). CAH, congenital adrenal hyperplasia

Prenatal diagnosis	*N *(%)	Postnatal diagnosis	*N* (%)
Hypospadias	52 (59)	Hypospadias	48 (54.5)
Clitoromegaly	21 (23.8)	Clitoromegaly	
		CAH	13 (14.7)
		Isolated transient	8 (9)
Micropenis	7 (7.9)	Micropenis	11 (12.5)
İsolated labial hypertrophy	5 (5.6)	Isolated labial hypertrophy	5 (5.6)
Uncertainty of sex determination	3 (3.4)	Uncertainty of sex determination	
		Persistent cloaca	2 (2.2)
		Normal 46, XY	1 (1.1)

Among hypospadias cases, chordee was documented in 11 (21.1%) fetuses on prenatal ultrasound. The penoscrotal angle was found to be less than 30° in five (45.4%) chordee cases, which were classified as proximal/penoscrotal hypospadias in the postnatal examination. Of 11 cases, the angle was over 30° in five (45.4%) cases classified as glandular/distal hypospadias (Table 3, Figure 5).

**Table 3:** Penoscrotal angle in hypospadias cases with chordee and classification of hypospadias in the postnatal period. The table presents the penoscrotal angle (a) measured in utero in different types of hypospadias. + indicates the penoscrotal angle in the corresponding column.

Penoscrotal angle (a, °)	Glanular distal hypospadias	Proximal penoscrotal hypospadias
27		+
55	+	
41		+
62	+	
45	+	
25		+
58	+	
23		+
64	+	
20		+
29		+

All fetuses with genital anomalies were evaluated with 3D imaging. Out of 17 clitoromegaly with labial hypertrophy, 12 (70.5%) had a *horseshoe* shape of enlarged labia in 3D views (Figures 3c-3d).

Thirteen out of 21 cases (61.9%) with clitoromegaly were diagnosed with congenital adrenal hyperplasia (CAH, 46, XX), while eight out of 21 cases (38%) were diagnosed with isolated transient clitoromegaly in the newborn period (Table 2). None of the mothers had received hormonal therapy in this group. All clitorises in the isolated transient clitoromegaly group returned to normal size by six months of age.

The overall prevalence of associated anomalies in our study was found to be 17% (15 cases, Table 1). Urogenital anomalies were identified in seven of the 88 genital defects, including bilateral hydronephrosis (*N *= 4), megacystis (*N *= 1), ovarian cyst (*N *= 1), and undescended testis (*N *= 1). Extremity anomalies in our cases included syndactyly (*N *= 1) and clinodactyly (*N *= 2). In two cases, cardiac anomaly (one atrioventricular septal defect and one aortic coarctation) was diagnosed. A central nervous system (CNS) anomaly (bilateral ventriculomegaly) was detected in one case, and an abdominal wall defect (omphalocele) was detected in another case.

Chromosome analysis revealed Trisomy 21 in one fetus (Table 1).

## Discussion

Fetal sex determination is the most frequently asked question by parents and has become a part of routine obstetric US. The accuracy of sex determination in the second trimester ranges between 92% and 100% in a normal population [8,9]. However, sex determination in fetuses with external genital anomalies may lead to a diagnostic challenge. Despite this, antenatal diagnosis of genital anomalies is still rare compared to other anomalies. In this study, the prevalence of genital anomalies was found to be 0.6% and the most common genital anomaly was hypospadias with a frequency of 59% in antenatal US. The DC between prenatal and postnatal diagnoses of external genital anomalies was 94.3%. This study is one of the studies with the largest number of genital anomalies diagnosed in the prenatal period and followed up postnatally.

Hypospadias is one of the most common and frequently missed anomalies in prenatal US [10]. The DC between prenatal and postnatal diagnoses of hypospadias in this study was 92.3%. There are different types of hypospadias, ranging from mild to severe forms depending on the location of the urethral opening. Mild hypospadias (glandular/distal hypospadias) is usually missed in the prenatal US; however, severe hypospadias (proximal/penoscrotal hypospadias) may be diagnosed more frequently, as in our study (27% mild hypospadias vs. 72.9% severe hypospadias). The *tulip sign* [4] and the presence of chordee may be useful to diagnose severe hypospadias. However, a prenatal sonographic finding that objectively indicates the severity of hypospadias has not been described so far. In this study, a penoscrotal angle was measured in cases with chordee to evaluate the severity of hypospadias. Five of the six cases whose penoscrotal angle was measured below 30° were diagnosed with severe hypospadias in the postnatal period. The PPV of the penoscrotal angle in diagnosing severe hypospadias was 83.3%.

Fetal clitoromegaly was the second most frequently diagnosed genital anomaly in this study, with a frequency of 23.8%. The DC between prenatal and postnatal diagnoses of clitoromegaly was 100%. When clitoromegaly is suspected, first, the pelvic organs of the fetus should be evaluated correctly. The fetal uterus can be recognized at 19 weeks of gestation [11]. The only part of the fetal uterus visible in the US is the cervix, which is more developed than the corpus throughout pregnancy [12]. When clitoromegaly is accompanied by hypertrophy of the labia, it can be confused with severe hypospadias. In our experience, the *three-line sign* indicating the labium majus, labium minus, and clitoris, which are used for female sex determination in the second trimester, can also be used in the differential diagnosis of clitoromegaly and severe hypospadias. In the second trimester, normal female genitalia appears as three straight lines parallel to each other, although in cases of clitoromegaly with labial hypertrophy, the outer two lines become concave and diverge (Figure 4a). 3D imaging can be useful for diagnosis because it better shows the anatomic structures. Approximately 70% of clitoromegaly with labial hypertrophy cases had enlarged labia in a *horseshoe* shape on 3D images. The *horseshoe* sign may be a diagnostic clue for labial hypertrophy on 3D imaging. Volumetric imaging also allows parents to be better oriented to the images and understand the severity of the anomaly. Approximately two out of every five clitoromegaly cases were transient clitoromegaly and disappeared in the postnatal period. Underlying factors for transient clitoromegaly have not been determined in recent papers; however, Zimmer et al. speculated that the relative immaturity of the adrenal glands in preterm newborns might cause transient clitoromegaly [5]. Other cases of clitoromegaly were accompanied by CAH, and accurate prenatal diagnosis was important in terms of early management in these babies.

Micropenis and isolated labial hypertrophy are less common anomalies with prevalences of 7.9% and 5.6%, respectively, among genital anomalies. Four of the 11 micropenis cases were misdiagnosed as hypospadias on the antenatal US. In this study, the sensitivity of antenatal US in the diagnosis of micropenis was found to be 73.3%. To the best of our knowledge, genital tubercle length is not very reliable in the differential diagnosis of hypospadias and micropenis [13]. More reproducible and reliable sonographic findings, such as 3D images of urethral canal position, may be available in the future with the improvement of imaging methods.

In our study, the rate of associated anomalies (17%) was between the reported rates of 7%-38% in the literature [13-14]. In our study, about half of the associated anomalies were urogenital anomalies. It has been stated in many articles that cloacal anomalies may accompany clitoromegaly [15,16]. In our study, the association of cloacal anomalies with clitoromegaly could not be determined and these anomalies were included in the *uncertainty of sex determination* group. In the presence of external genital anomalies, a complete and systematic antenatal US should be made in terms of associated anomalies.

There are some limitations of this study. Since there was no standardized description of the external genital organs and no objective measurement of the genital tubercle in the literature, external genitalia could be evaluated subjectively. The absence of a definitive genetic and hormonal evaluation protocol in the prenatal period was another limitation of this study. However, the main purpose of this study was to provide clues for prenatal diagnosis of genital anomalies.

## Conclusions

Prenatal diagnosis of external genital anomalies is of great importance for both clinicians and parents in terms of accurate determination of fetal gender and early management. The DC between prenatal and postnatal diagnoses of external genital anomalies is high in this study. Diagnostic clues such as the *horseshoe sign* of the labia in 3D images should be used in the diagnosis of clitoromegaly with labial hypertrophy. In male fetuses with chordee, a penoscrotal angle below 30 degrees may indicate that hypospadias is serious. Further studies are needed to confirm the value of prenatal sonographic clues in diagnosing and determining the severity of external genital anomalies.

## References

[1] van der Horst HJ, de Wall LL (2017). Hypospadias, all there is to know. Eur J Pediatr.

[2] Källén B, Bertollini R, Castilla E (1986). A joint international study on the epidemiology of hypospadias. Acta Paediatr Scand Suppl.

[3] Yu X, Nassar N, Mastroiacovo P (2019). Hypospadias prevalence and trends in international birth defect surveillance systems, 1980-2010. Eur Urol.

[4] Meizner I (2002). The 'tulip sign': a sonographic clue for in-utero diagnosis of severe hypospadias. Ultrasound Obstet Gynecol.

[5] Zimmer EZ, Blazer S, Blumenfeld Z, Bronshtein M (2012). Fetal transient clitoromegaly and transient hypertrophy of the labia minora in early and mid pregnancy. J Ultrasound Med.

[6] Cheikhelard A, Luton D, Philippe-Chomette P (2000). How accurate is the prenatal diagnosis of abnormal genitalia?. J Urol.

[7] Jamigorn M, Phupong V (2017). Nomograms of the whole foetal adrenal gland and foetal zone at gestational age of 16-24 weeks. J Obstet Gynaecol.

[8] Emerson DS, Felker RE, Brown DL (1989). The sagittal sign. An early second trimester sonographic indicator of fetal gender. J Ultrasound Med.

[9] Odeh M, Granin V, Kais M, Ophir E, Bornstein J (2009). Sonographic fetal sex determination. Obstet Gynecol Surv.

[10] Sparks TN (2021). Hypospadias. Am J Obstet Gynecol.

[11] Pinson K, Melber DJ, Nguyen NH (2023). The development of normal fetal external genitalia throughout gestation. J Ultrasound Med.

[12] Lépinard C (2006). Assessment of the female fetal pelvis using conventional two-dimensional ultrasound. Ultrasound Obstet Gynecol.

[13] Fuchs F, Borrego P, Amouroux C (2019). Prenatal imaging of genital defects: clinical spectrum and predictive factors for severe forms. BJU Int.

[14] Epelboym Y, Estrada C, Estroff J (2017). Ultrasound diagnosis of fetal hypospadias: accuracy and outcomes. J Pediatr Urol.

[15] Chadha R, Kothari SK, Tanwar US, Gupta S (2001). Female pseudohermaphroditism associated with cloacal anomalies: faulty differentiation in the caudal developmental field. J Pediatr Surg.

[16] Macarthur M, Mahomed A (2006). Rare association of female pseudohermaphroditism, phallic urethra, and posterior cloaca. J Pediatr Surg.

